# Continuous dose-intense temozolomide and cisplatin in recurrent glioblastoma patients

**DOI:** 10.1097/MD.0000000000006261

**Published:** 2017-03-10

**Authors:** Yu Wang, Xiangyi Kong, Yi Guo, Renzhi Wang, Wenbin Ma

**Affiliations:** a Department of Neurosurgery, Peking Union Medical College Hospital, Chinese Academy of Medical Sciences, Beijing, PR China; b Department of Anesthesia, Critical Care and Pain Medicine, Massachusetts General Hospital, Harvard Medical School, Harvard University, Boston, MA.

**Keywords:** cisplatin, dose-intense, glioblastoma, recurrent, temozolomide

## Abstract

In glioblastoma multiforme (GBM), both temozolomide (TMZ) and cisplatin are very active at various toxic levels. Previous studies demonstrated that cisplatin with the standard regimen of TMZ is active in patients suffering from recurrent GBM, generating a moderate level of toxicity. Also, continuous dose-intense TMZ is a helpful therapy for patients with recurrent GBM. We have conducted a research to evaluate the security and effectiveness of cisplatin with constant dose-intense TMZ for reduplicative GBM. The time to progression (TTP) and progression-free survival (PFS) at 6 months (PFS-6) was the major end point. Toxicity, overall survival, and response are the secondary end points. GBM patients who suffered from progression or relapse after surgery, radiotherapy, and chemotherapy were qualified. Cisplatin 40, 30, and 30 mg were given on days 1, 2, and 3 before the corresponding TMZ doses, respectively. Without interruption, TMZ was given at a dose of 50 mg/m^2^ on everyday basis (dose-intense) until development or progression of unacceptable side effects. A cycle was defined as 28 days. Response Assessment in Neuro-Oncology criteria were utilized to evaluate the response. Twenty-seven patients in total (median Karnofsky performance status—80, ranging from 60 to 100; average age—56 years, ranging from 24 to 78 years) were accrued in the research. PFS-12 was 11.1% (95% confidence interval [CI], −0.7% to 22.9%), and PFS-6 was 37% (95% CI, 18.8%–55.2%). Twenty-three weeks was the median TTP (95% CI, 17–29 weeks). In the 27 evaluative patients, 6 partial responses were observed with an overall response rate of 22.2% (95% CI, 6.5%–37.9%), while no complete response was obtained. Toxicity was mostly of grades 1 to 2 amongst 116 therapy cycles. Hematological and gastroenterological toxicities were the major limiting side effect found in the research. One patient has received leukopenia World Health Organization grade 4 at cycle 5 during her treatment. Eight percent of patients had grades 3 to 4 vomiting/nausea. As a valuable therapeutic option, the innovative cisplatin with continuous dose-intense regimen of TMZ incurs an acceptable level of toxicity and shows active performance in patients with recurrent GBM.

## 1 Introduction

As one of the most deadly neoplasms, glioblastoma multiforme (GBM) continues to be regarded as incurable and universally fatal. The prognosis for patients suffering from recurrent GBM is even poorer, with an average survival time of around half a year. The clinical experiments of combination chemotherapy or single-agent in this patient population yield the response rate from 5% to 20%.^[1]^ With a comprehension of growth factor pathway and development in cancer biology, clinical experiments on the inhibitors of growth factor have been conducted. However, the findings are not satisfactory, and no progress has been seen in the survival rate or response rate.^[2–5]^

Present standardized treatment of primary GBM is neurosurgery followed by 30 times of 2-Gy irradiation combined with daily temozolomide (TMZ) chemotherapy (75 mg/m^2^), with 6 subsequent monthly adjuvant cycles of TMZ chemotherapy (150–200 mg/m^2^ daily for 5 days). Under such a therapeutic schedule, the median overall survival (OS) could reach 14.6 months.^[6]^ The epigenetic silencing of the deoxyribose nucleic acid (DNA) repair enzyme O6-methylguanine-DNA-methyltransferase (MGMT, which can repair the DNA damage caused by TMZ and other alkylating agents) in tumor tissues is regarded as one of the reasons for the effectiveness of TMZ.^[7,8]^ As such, this enzyme can be used to predict the response to TMZ. Less MGMT proteins are generated by tumor cells with a methylated MGMT promoter, so the patients can benefit more from TMZ.^[9]^

The methylation proportion for MGMT promoter in GBM patients is approximately 40%.^[9]^ A potential means to surpass an unmethylated promoter is the usage of continuous TMZ below the maximal tolerance dose (prolonged low doses) in order to deplete MGMT and sensitize tumor cells to TMZ.^[9]^ On a constant foundation, dose-intense TMZ might also restrict the activity and mobilization of circulating endothelial precursors and marrow-derived cells as well as prevent the recovery of endothelial cells, therefore making contribution to the antiangiogenic activity.^[10]^ The traditional 5-day TMZ regimen delivers from 750 to 1000 mg/m^2^ on monthly basis. Oppositely, protracted TMZ at 50 mg/m^2^ every day, applied in the present research, provides an intense dose of 1400 mg/m^2^ for the per 28-day period. This stands for 1.4 to 1.9 more TMZ dose than in the regular 5-day regimen of TMZ.^[11]^ Recently, interesting results show that protracted dose-intense TMZ administration yielded an overall response rate (ORR) of 41% in people suffering from recurrent glioblastoma with 75 to 100 mg/m^2^ on daily basis for 6 to 7 weeks. This response rate appeared better than that in similar groups of standard 5-day regimen.^[12]^

The most important predictor of efficacy was MGMT status. Regardless of the size of the dose, the rate of PFS-6 showed higher efficacy in patients with methylated MGMT promoter than that in unmethylated group.^[7,9]^ Studies have shown in vitro that cisplatin was able to decrease the activity of MGMT, indicating that the single-agent TMZ activity could be increased by cisplatin.^[13]^ Furthermore, the rationale for such combination stemmed from preclinical studies showing no overlapped toxic profiles when cisplatin has a synergistic effect with TMZ, thereby allowing the administration of both drugs in phase II clinical trials on full does.^[13]^ In Silvani study, standard TMZ administration [200 mg/m^2^ on days 2–6 once four weeks (q4wk)] and cisplatin (40 mg/m^2^, on days 1 and 2 q4wk) increased PFS-6 to 35%.^[14]^ This is comparable to those obtained combining TMZ plus other drugs and compares favorably with those obtained using a standard schedule of TMZ alone. In the present study, we explored the efficacy and the toxicity of the combination of cisplatin with continuous dose-intense TMZ in patients with recurrent GBM. As we did not see such kind of studies by PubMed and EMBASE, we believe this is the first one.

## 2 Materials and methods

### 2.1 Study population

Eligibility criteria: 18 to 70 years old, histologic diagnosis of GBM, platelets (≥100,000/μL), normal baseline counts for neutrophils (≥1500/μL), Karnofsky performance status (KPS) ≥60, creatinine and bilirubin levels ≤1.25 times of the upper limits of normal, alkaline phosphatase and transaminases degrees ≤1.5 times of the upper limits of normal, and prior surgery followed by standard radiation therapy (60 Gy/30 fractions). The progression of disease and the evidence of relapse had to be confirmed using neuroimages with gadolinium-enhanced magnetic resonance imaging (MRI). Only patients with disease progression (PD) in 2 MRIs separated by not less than 1 month, with at least 1 enhancing measurable lesion diameter of ≥2 cm, were accrued in our research. Breast feeding or pregnant patients were regarded ineligible. All the patients gave their fully informed consent to participate in the research by signing a form. The approval from the Institutional Review Board of Peking Union Medical College Hospital, China, has been obtained. The study was carried out based on the rules of Good Clinical Practice^[15]^ and World Medical Association Declaration of Helsinki.^[16]^ The patient characteristics were shown in Table 1.

**Table 1:**
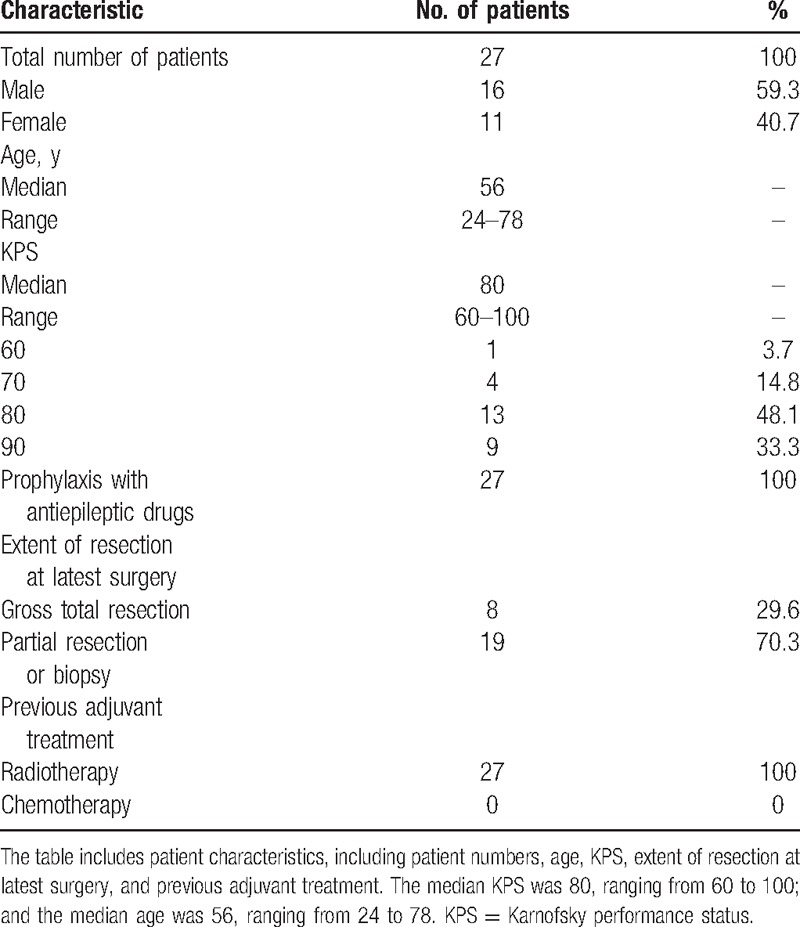
Patient characteristics.

### 2.2 Treatment schedule

**Figure 1:**
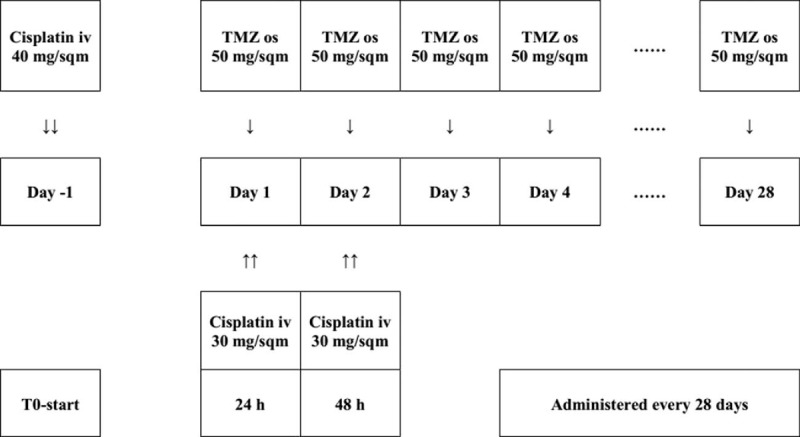
The 28-day scheme of cisplatin and continuous dose-intense temozolomide.

We used a 28-day scheme of cisplatin and TMZ association (Fig. 1). A total of 100 mg of cisplatin was used in each cycle. On day 1, cisplatin 40 mg, after the dissolution in 500 mL 0.9% saline, was administered intravenous injection (i.v.) over 1 hour before the first TMZ dose; on days 2 and 3, cisplatin 30 mg was administered i.v. before the second and third TMZ doses, respectively. Preceding cisplatin, patients received prophylactic parenteral antiemetics, consisting of 8 mg i.v./ondasteron, 100 mg i.v./alizapride and i.v./dexamethasone (8 mg). TMZ was given at a dose of 50 mg/m^2^ daily continuously (dose-intense), without interruption, until progression or development of unacceptable side effects. Patients were required to fast for at least 2 hours before and after each TMZ dose. One cycle was defined as 28 days. The treatment was held for one of the subsequent occurred events: grade ≥4 nonhematologic toxicity, grade ≥2 central nervous system hemorrhage, documented tumor progression, noncompliance with researching guidelines, or voluntary withdrawal. When toxicity was resolved, the treatment was restarted. As clinically indicated, the dose of TMZ could be decreased by 25% in accordance with specific conditions. However, if further reduction was needed, patients would be removed from the study. A comprehensive chemistry panel was performed every month, and the complete blood counts were repeated every week. Response was assessed every 8 weeks, before every other cycle using gadolinium-enhanced MRI; the standardized definitions of response were adapted from the standard of the Response Assessment in Neuro-Oncology (RANO) criteria^[17]^ (Table 2). Recombinant human granulocyte colony-stimulating factor was prescribed to prevent or treat neutropenia caused by chemotherapy according to laboratory findings.

**Table 2:**
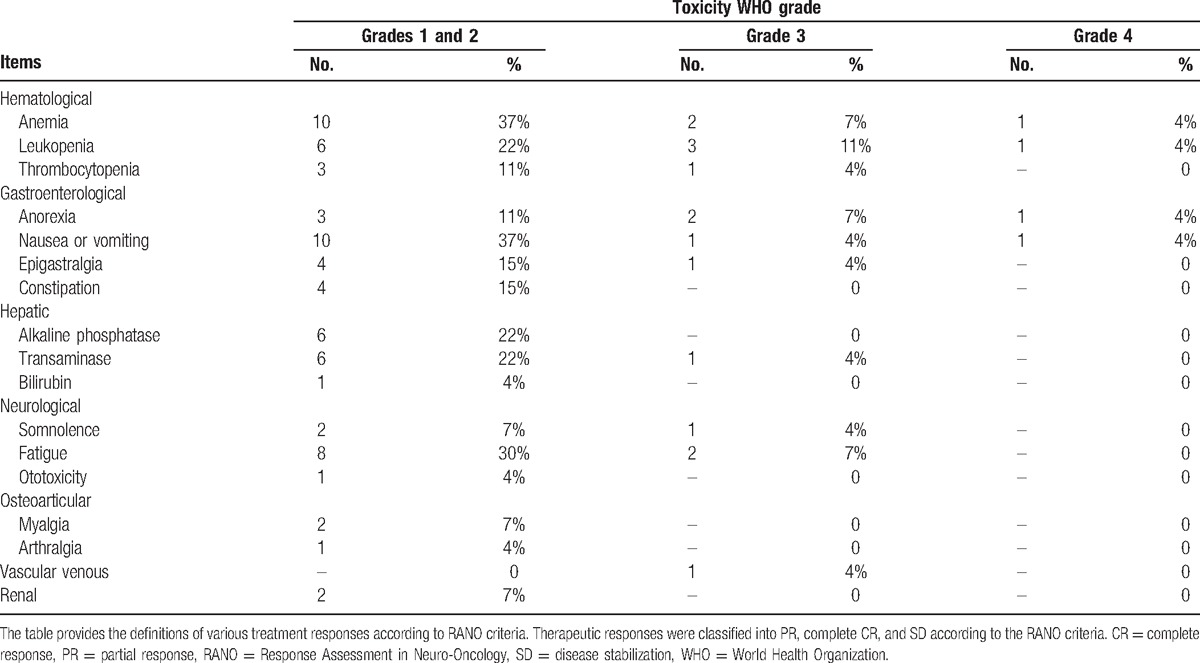
Toxicity in 27 patients.

### 2.3 Efficacy measures

Time to progression (TTP) from the start of chemotherapy to progression or exit from the study for any reason was evaluated. The OS time was calculated from the beginning of chemotherapy to death. The statistical analysis involved all data from the 27 patients. OS, TTP, and PFS-6 were calculated by virtue of Kaplan–Meier method^[18]^; differences in OS and TTP were assessed for statistical importance by the log-rank test.^[19]^ Patients were evaluated for response according to clinical and neurologic examinations (performed monthly before each cycle) and MRI or positron emission tomography–computed tomography (PET-CT) neuroimaging performed every 2 cycles, or earlier if clinically indicated, based on the RANO criteria.^[17]^ Neurologic conditions were evaluated in consideration of symptoms and signs possibly related to tumor progression, in comparison to the prior examinations; every change in the daily dosage of corticosteroids was kept track of.

Therapeutic responses were classified into partial (PR), complete (CR), and disease stabilization (SD) according to the RANO criteria^[17]^ (Table 2) only if they were constant on successive brain MRIs with at least 4 weeks’ interval. MRI was conducted with a research-particular brain tumor protocol including T1-weighed image, T2-weighed image, fluid attenuation inversion recovery image, diffusion-weighted imaging, and perfusion-weighted imaging. Patients were removed from the study if they retracted their consents, encountered severe toxicity, or had progressive disease. Patients who interrupted treatment before the first radiologic assessment were regarded evaluative for only toxicity rather than response.

### 2.4 Statistical analysis

The major end point was PFS-6. Descriptive approaches were used to report the baseline demographics, toxicity, and response rate. OS, PFS-6, and TTP were calculated based on the Kaplan–Meier and log-rank test by means of IBM SPSS Statistics 20.0 (International Business Machines Corporation, Armonk, New York, United States). Parameters related to OS and therapeutic response were KPS, age, and time interval between the end of radiation therapy and relapse.

Multivariate analysis based on the Cox model was applied in the evaluation of independent prognostic factors and was conducted on variates with *P* < 0.05 at univariate analysis.

## 3 Results

### 3.1 Demographics

From June 2013 to January 2015, 27 patients (16 males) were included in this research. All patients were assessable for response and toxicity. The clinical and demographic features of them are listed in Table 1. The median KPS was 80, ranging from 60 to 100; and the median age was 56, ranging from 24 to 78. Twenty-four patients had experienced 1 surgery and 3 experienced more than 1. Based on the findings at postoperative neuroimaging or the neurosurgeon's impression, the last surgery was regarded macroscopically radical in 8 people, accounting for 29.6%. All patients had undergone radiotherapy and chemotherapy treatment. Antiepileptic prophylaxis medication, Depakine (Sanofi Pharmaceutical Company, Hangzhou, Zhejiang Province, P.R. China) was given to all the 27 patients. The median follow-up period reached 16.77 months, ranging from 2.8 to 26.6 months.

### 3.2 Disease progression

**Figure 2:**
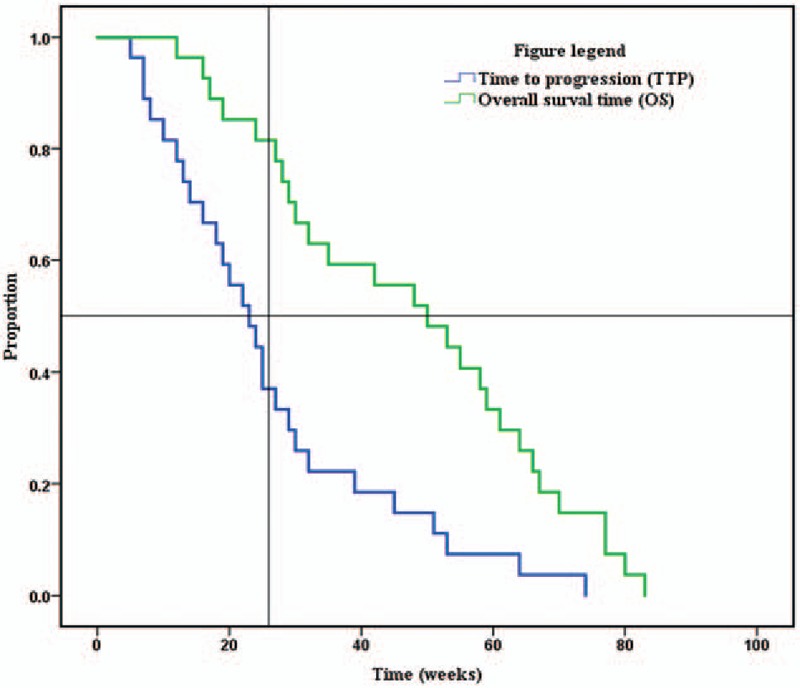
Survivorship curve for the time to progression and overall survival of the 27 patients.

Considering all 27 patients, median TTP was 23 weeks (95% confidence interval [CI], 17–29 weeks); PFS-6 and PFS-12 were 37% (95% CI, 18.8%–55.2%) and 11.1% (95% CI, −0.7% to 22.9%), respectively (Table 3, Fig. 2). Ten of the 27 patients were free of PD at 6 months; this finding, confirmed by an independent centralized review, surpassed the objective of 37%, reported in Brandes et al^[20]^ study (cisplatin and standard TMZ regimen). Responders (CR + PR) had a higher TTP (mean, 40.83 weeks; 95% CI, 22.9–58.8 weeks) than patients with progressive disease (mean, 10.7 weeks; 95% CI, 6.9–14.5 weeks; Chi-square = 12.74, *P* < 0.0001). Patients with SD had a mean TTP of 24 weeks (range, 10–53 weeks; 95% CI, 21.6–26.4 weeks). No significant difference in TTP was found between patients with CR and PR and those with SD (Chi-square = 2.43, *P* = 0.12). No statistical differences for KPS, age, surgery type, the time between the beginning of chemotherapy and the first surgery, as well as the time between the beginning of radiotherapy and the beginning of chemotherapy were shown in the TTP assessment using the log-rank test. The stabilization or response obtained from the treatment was the only predictive factor for the progression (*P* < 0.001).

**Table 3:**
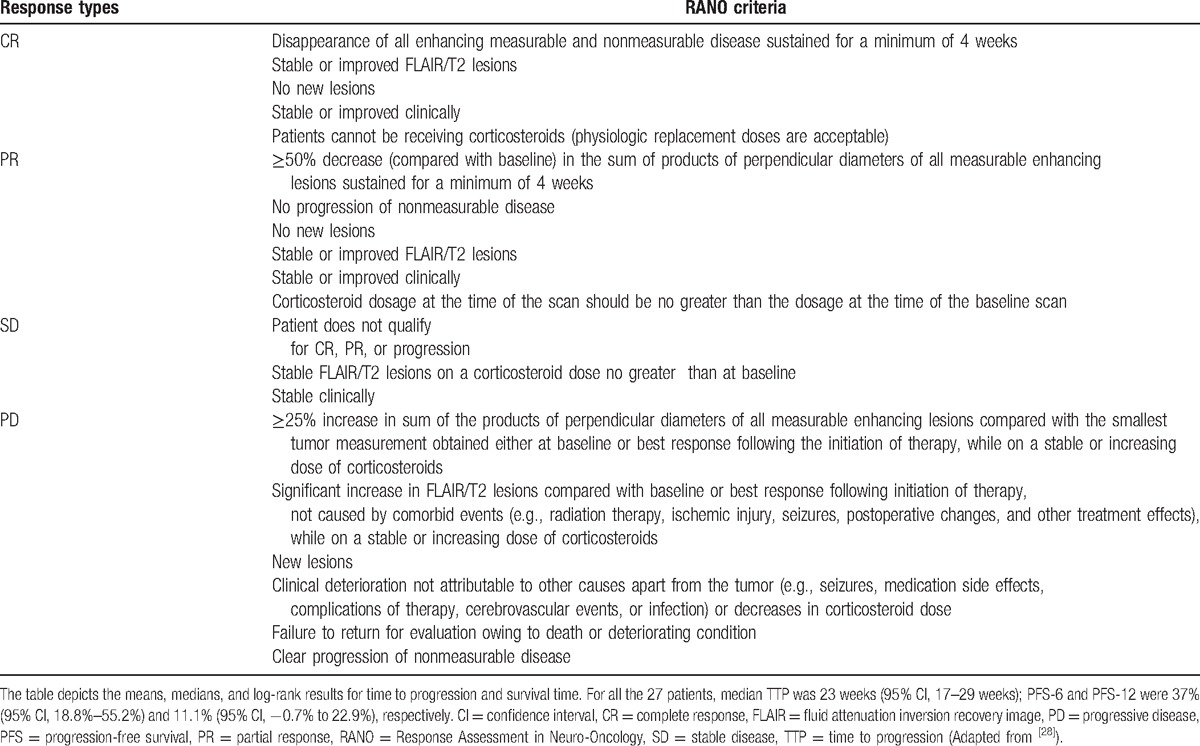
Definitions of various treatment responses according to Macdonald criteria.

### 3.3 Overall survival

In spite of the possible influence of the second-line treatments, median OS in the population researched was 50 weeks (95% CI, 31.3–68.7 weeks), measured from the start of the chemotherapy. The OS rates at 6 and 12 months were 81.5% (95% CI, 66.8%–96.2%) and 48.1% (95% CI, 29.3%–66.9%), respectively (Table 3, Fig. 2). There was no prognostic factor considerably related to the survival.

### 3.4 Response

**Figure 3:**
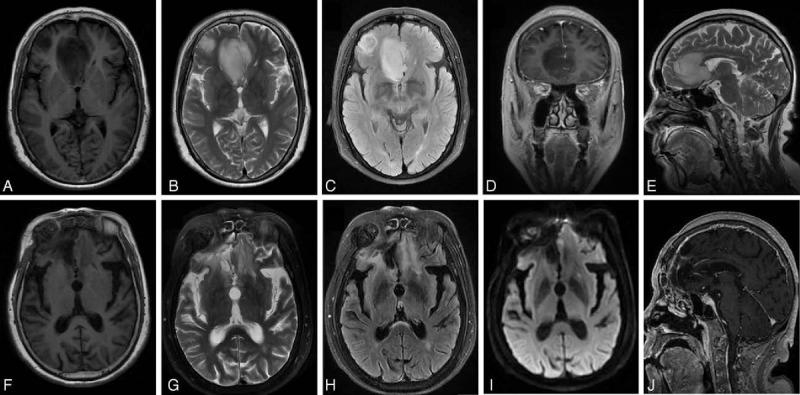
Brain magnetic resonance imaging showed a partial response of glioblastoma in a 46-year-old male patient before surgery and after 7 cycles of chemotherapy. (A) Preoperative, axial view, T1-weighed image (T1WI). (B) Preoperative, axial view, T2-weighed image (T2WI). (C) Preoperative, axial view, fluid attenuation inversion recovery image (FLAIR). (D) Preoperative, coronal view, contrast-enhanced T1WI. (E) Preoperative, sagittal view, T2WI. (F) Postoperative, axial view, T1WI. (G) Postoperative, axial view, T2WI. (H) Postoperative, axial view, FLAIR. (I) Postoperative, axial view, diffusion-weighted imaging. (J) Postoperative, sagittal view, contrast-enhanced T1WI.

**Figure 4:**
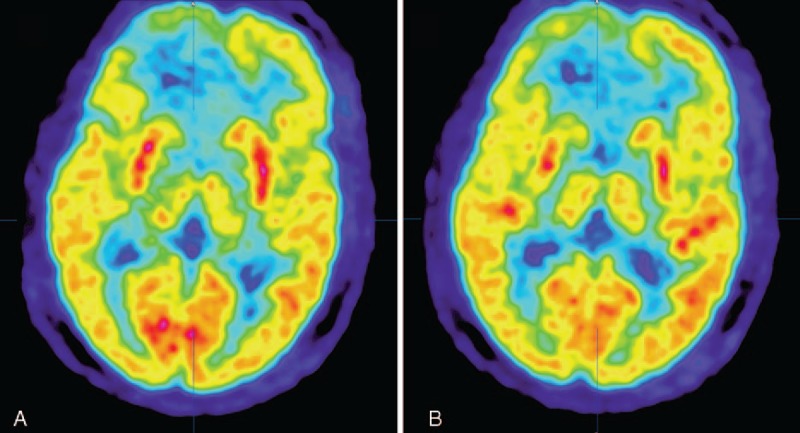
Preoperative brain positron emission tomography–computed tomography (PET-CT) of the patient described in Fig. 3. Because of the formation of necrosis inside the tumor, low 18F-fluorodeoxyglucose uptake in the tumor area was shown. We did not perform another PET-CT examination after the surgery.

In the 27 assessable patients, no CR was observed, and 6 PRs were obtained, with an ORR of 22.2% (95% CI, 6.5%–37.9%) (Figs. 3 and 4). All radiologic responses were confirmed by an independent centralized review, and stable or decreased steroid dosage was confirmed in all patients at the time of recording response. The median OS and median TTP of response patients (CR + PR) were 61 (range, 35–83 weeks; 95% CI, 52.6–69.4 weeks) and 29 weeks (range, 19–74 weeks; 95% CI, 23.0–35.0 weeks), respectively. The median OS and median TTP of SD, achieved in 14 patients (51.9%; 95% CI, 31%–63%), were 53 (range, 24–77 weeks; 95% CI, 43.8–62.2 weeks) and 24 weeks (range, 10–53 weeks; 95% CI, 21.6–26.4 weeks).

No correlation was found between age (*P* = 0.79, Student *t* test), KPS (*P* = 0.618, Mann–Whitney test), and interval between the end of radiotherapy and start of the present protocol (*P* = 0.24, Mann–Whitney test).

### 3.5 Toxicity

Based on the common toxicity standard of National Cancer Institute (version 3.0), all toxicities have been graded; 116 therapy cycles in total were given to all the patients. An average of 4.3 cycles was given to every patient, ranging from 1 to 8. Toxicity (Table 4) was mostly of grades 1 and 2. Hematological toxicity was the major limiting side effect in this research (Table 4). It resulted in infused condensed erythrocyte in 1 patient. One patient experienced leukopenia (grade 4) at her fifth treatment cycle and was hospitalized due to infection. There was no hemorrhagic event. Gastroenterological toxicity was another main side effect, including anorexia, nausea, vomiting, epigastralgia, and constipation. Grades 1 to 2 vomiting/nausea was found in 37% of patients, and grades 3 to 4 was found in 8% patients. Ten patients suffered from fatigue, and 2 of them was grade 3. This symptom did not seem to be related to the anemia, but a direct consequence of the chemotherapy administration. Mild (grade 1) ototoxicity in 1 patient was observed. The lab evaluations of liver and renal function demonstrated only slight abnormalities. Because of the chemotherapy-related episodes of grades 3 to 4 toxicity, 8 patients needed a dosage reduction to 75% or less.

**Table 4:**
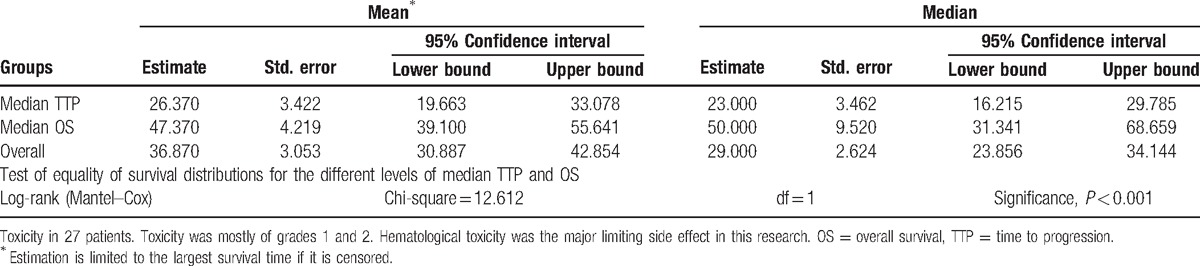
Means, medians, and log-rank results for time to progression and survival time.

## 4 Discussion

**Figure 5:**
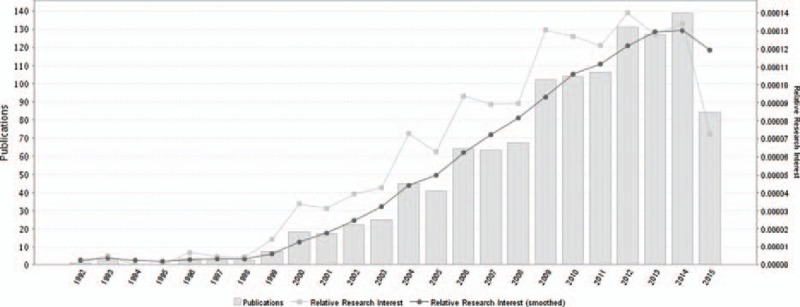
A timeline of the publications related to temozolomide for gliomas.

**Figure 6:**
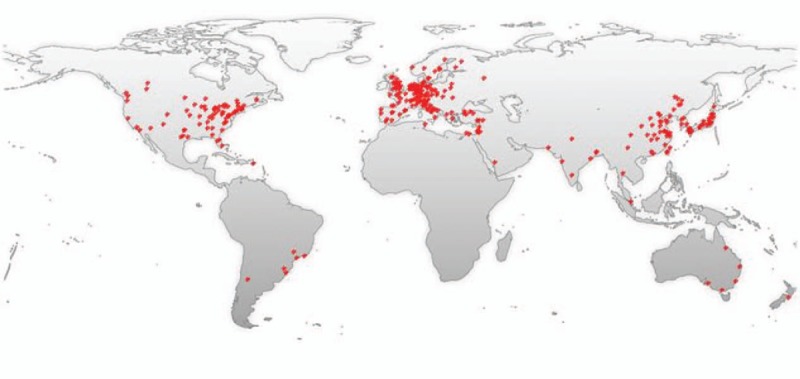
A world map with the global distribution of publications about temozolomide for gliomas based on the analysis of their geolocational data.

Newly diagnosed glioblastoma is now commonly treated with surgery, if feasible, or biopsy, followed by radiation and concomitant and adjuvant TMZ.^[21]^ The number of relevant literatures about the efficacy of TMZ on gliomas in each year shows a general tendency to increase over time. A timeline of the related publications is available as Fig. 5. Through making a world map based on the geolocational data of global distribution of related publications, the countries that the publications are from are primarily concentrated in North America, Europe, and East Asia (Fig. 6). The present standard of treatment for newly diagnosed GBM was set up in 2005 (Stupp schedule, concurrent TMZ [75 mg/m^2^/d for no more than 7 weeks] and radiotherapy followed by 6 cycles of adjuvant chemotherapy [150–200 mg/m^2^ on 5-day therapy every 28 days]).^[6]^ However, the optimal treatment schedule for recurrent GBM has not been established. Dismal outcomes with PFS-6 of 9% to 21%, objective response rates (ORRs) of 5% to 6%, and median OS of 6 to 7 months were reported in previous clinical studies in recurrent GBM.^[22–24]^ With the exception of bevacizumab, therapies with a great number of nontargeted and targeted agents have been attempted without success. On the basis of better ORRs of 20% to 26% observed in 2 phase II experiments, phase II gained accelerated approval of FDA for recurrent GBM.^[24,25]^ Nonetheless, the OS is modest (median, 8–10 months), and the responses are short-lived (median, 4 months).^[24]^

Except for the standard TMZ schedule, there is increasing interest in other TMZ regimens, especially regimens with continuous dose-intense TMZ administration.^[10–12,24,26–28]^ Protracted TMZ regimen is likely to reduce the influence of unmethylated MGMT promoter, a critical cause for TMZ resistance, and can offer a higher delivery dose every month. In addition, preclinical studies showed that continuous dose-intense TMZ administration also has somewhat antiangiogenic activity. On the other hand, currently, as the object of lots of clinical experiments aiming to make improvements on the basis of Stupp schedule or to discover new regimens to further minimize the chemoresistance, TMZ has been combined with several other cytostatic or cytotoxic agents. In the past 10 years, a plethora of phase I and II studies have explored the safety and efficacy of TMZ combined with interferon, nitrosoureas, and bevacizumab and some miscellaneous/conventional chemotherapeutics including pegylated doxorubicin, irinotecan, capecitabine, sorafenib, and cisplatin for progressive or recurrent GBM.^[1,4,17,25]^

In Graviani study, which was halted early due to unaccepted side effects of granulocytopenia and thrombocytopenia, the combination of fotemustine and TMZ was explored in 10 people suffering from recurrent GBM following chemoradiotherapy.^[29]^ The author believed that such combination does not deserve further research.^[29]^ Also, TMZ was once combined in a phase II trial with the epidermal growth factor receptor antagonist, afatinib (40 mg/d), resulting in the PFS-6 of 10% (23% for TMZ alone [*P* = 0.59] and 3% for afatinib alone [P = 0.008]).^[30]^ Severe toxic and side effects (grade ≥3) for this regimen were mainly nonhematologic (such as skin allergy, emesis, and diarrhea).^[30]^

A phase II clinical trial of combining TMZ with cisplatin in patients with recurrent high-grade glioma was reported by Silvani et al^[14]^. Cisplatin (40 mg/m^2^, intravenous injection) was given in the first 2 days. From days 2 to 6 (beginning 24 hours after the first dosage of cisplatin), TMZ (200 mg/m^2^) was given as an individual oral daily dosage, and the cycle was rotated every 4 weeks. The PR and SD accounted for 18.8% and 39.9%, respectively. No CR was observed. PFS-6, PFS-12, and median TTP for GBM were 35%, 13.8%, and 33 weeks, respectively. In the same year, Brandes et al^[20]^ also published a similar study containing 50 recurrent GBM patients. On day 1, cisplatin (75 mg/m^2^, intravenous injection) was administered. On days 2 to 6, TMZ 130 mg/m^2^ bolus followed by 9 doses of 70 mg/m^2^ every 12 hours. The cycle was also repeated every 4 weeks. In this study, PFS-6, PFS-12, and median PFS were 34%, 4% and 18.4 weeks, respectively. Amongst the 49 assessable patients, 9 PRs and 1 CR were seen. The ORR was 20.4%.^[20]^

In our trial, a dose-intense TMZ regimen (TMZ was given at a dose of 50 mg/m^2^ daily continuously for 28 days as a cycle) was combined with cisplatin administration on days 1 (40 mg), 2 (30 mg), and 3 (30 mg). The combination was well tolerated with an amount of hematological toxicity that is not considerably different from the data observed after single TMZ or cisplatin chemotherapy. The principal nonhematological manifestations of toxicity were nausea and vomiting. The increased duration and incidence of the toxicity and side effects was expected compared to the single TMZ use and was thought to correlate with cisplatin's inherent emetogenic potentials. A possible drawback of the administration of a second round of cisplatin-based chemotherapy was the potential intrinsic neurotoxicity. However, toxicity was mostly of grades 1 to 2 (Table 4). No patients encountered TMZ dosage reduction more than 25%, thus no one was removed from the study.

When this present regimen was given to the chemotherapy-naive patients with recurrent GBM, a PFS-12 of 11.1% (95% CI, −0.7% to 22.9%) and a PFS-6 of 37% (95% CI, 18.8%–55.2%) were obtained. The median TTP was 23 weeks (95% CI, 17–29 weeks). One hundred sixteen cycles in total were administered with a median for every patient of 4, ranging from 1 to 8. The adverse events of toxicity were mild (mostly grades 1–2). The outcomes turned out to be favorable with or at least be comparable to those seen with standard TMZ schedule (Stupp schedule) and cisplatin or other chemotherapeutic drugs (Table 5). However, the adjustments for the distribution of known prognostic factors cannot be performed in nonrandomized forms. Furthermore, prior radiochemotherapy exposure or surgery situation may reduce PFS-6 and chemosensitivity in these experiments mentioned. Evaluating the results of our nonrandomized phase-II study, an important bias in their interpretation could derive from the inclusion of a population with favorable prognostic factors. However, this inadequacy is common to several reported studies. It might be worth stressing that the inclusion criteria in our group of patients were relatively strict to reduce the bias as much as possible. The progression of disease and the evidence of relapse had to be confirmed using enhanced MRI. Only patients with PD in 2 MRIs separated by not less than 1 month, with at least 1 enhancing measurable lesion diameter of ≥2 cm, were accrued in our research.

**Table 5:**
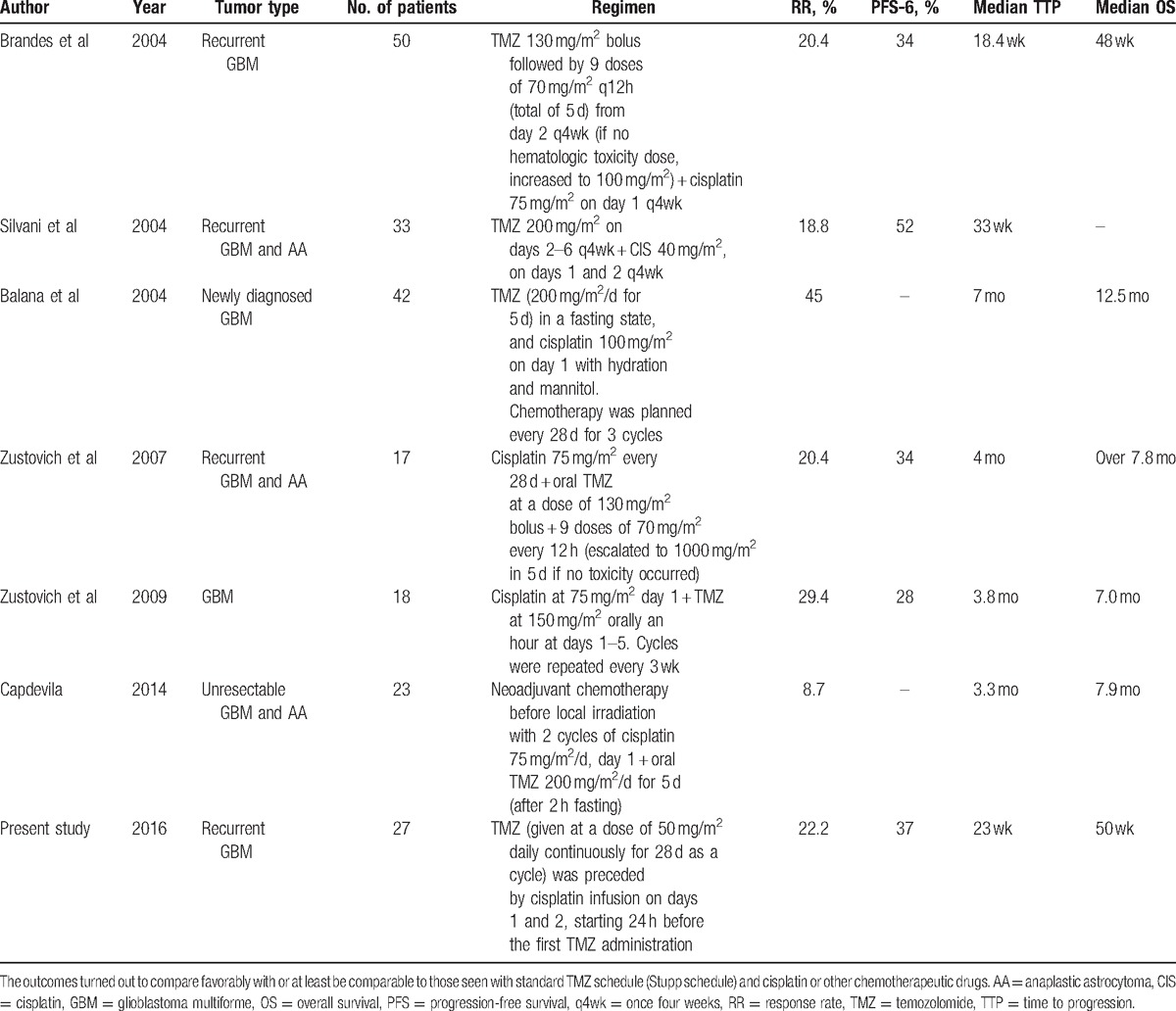
Chemotherapy studies of TMZ and platinum drugs in gliomas.

## 5 Conclusion

The use of chemotherapy in primary and recurrent glioblastoma chemotherapy is limited by the acquired or intrinsic cells’ resistance to the drugs. An improved understanding of the mechanisms of resistance and action to platinum compounds and TMZ is needed. At present, the results of dose-intense TMZ and cisplatin are promising, even though they relate to a small nonrandomized group of patients. This regimen appears to be an active one in terms of progression delay and seems to be more effective than the regimen of cisplatin and standard TMZ schedule. Since there was no available definitive data on the results of patients with the single use of TMZ to our knowledge, we cannot confirm just yet that the promising outcomes obtained in our present study are due to the peculiar regimen of dose-intense TMZ use, to the cisplatin use, or both. Thus, more randomized clinical trials on a wide cross-section of patient population are required with the aim of improving the dismal prognosis of recurrent GBM patients and overcoming the chemoresistance.

## Acknowledgments

We would like to thank our colleagues from the Department of Neurosurgery, Peking Union Medical College Hospital; Chinese Academy of Medical Sciences; and Peking Union Medical College.
